# Upper Gastrointestinal Symptoms Predictive of Candida Esophagitis and Erosive Esophagitis in HIV and Non-HIV Patients: An Endoscopy-Based Cross-Sectional Study of 6011 Patients

**DOI:** 10.1097/MD.0000000000002138

**Published:** 2015-10-30

**Authors:** Yuta Takahashi, Naoyoshi Nagata, Takuro Shimbo, Takeshi Nishijima, Koji Watanabe, Tomonori Aoki, Katsunori Sekine, Hidetaka Okubo, Kazuhiro Watanabe, Toshiyuki Sakurai, Chizu Yokoi, Akio Mimori, Shinichi Oka, Naomi Uemura, Junichi Akiyama

**Affiliations:** From the Department of Gastroenterology and Hepatology, National Center for Global Health and Medicine, Tokyo (YT, NN, TA, KS, HO, KW, TS, CY, JA); Ohta Nishinouchi Hospital, Fukushima (TS); Division of AIDS Clinical Center, National Center for Global Health and Medicine (TN, KW, SO); Division of Rheumatic Diseases, National Center for Global Health and Medicine, Tokyo (AM); and Department of Gastroenterology and Hepatology, National Center for Global Health and Medicine, Kohnodai Hospital, Chiba, Japan (NU).

## Abstract

Upper gastrointestinal (GI) symptoms are common in both HIV and non-HIV-infected patients, but the difference of GI symptom severity between 2 groups remains unknown. Candida esophagitis and erosive esophagitis, 2 major types of esophagitis, are seen in both HIV and non-HIV-infected patients, but differences in GI symptoms that are predictive of esophagitis between 2 groups remain unknown. We aimed to determine whether GI symptoms differ between HIV-infected and non-HIV-infected patients, and identify specific symptoms of candida esophagitis and erosive esophagitis between 2 groups.

We prospectively enrolled 6011 patients (HIV, 430; non-HIV, 5581) who underwent endoscopy and completed questionnaires. Nine upper GI symptoms (epigastric pain, heartburn, acid regurgitation, hunger cramps, nausea, early satiety, belching, dysphagia, and odynophagia) were evaluated using a 7-point Likert scale. Associations between esophagitis and symptoms were analyzed by the multivariate logistic regression model adjusted for age, sex, and proton pump inhibitors.

Endoscopy revealed GI-organic diseases in 33.4% (2010/6.011) of patients. The prevalence of candida esophagitis and erosive esophagitis was 11.2% and 12.1% in HIV-infected patients, respectively, whereas it was 2.9% and 10.7 % in non-HIV-infected patients, respectively. After excluding GI-organic diseases, HIV-infected patients had significantly (*P* < 0.05) higher symptom scores for heartburn, hunger cramps, nausea, early satiety, belching, dysphagia, and odynophagia than non-HIV-infected patients. In HIV-infected patients, any symptom was not significantly associated with CD4 cell count. In multivariate analysis, none of the 9 GI symptoms were associated with candida esophagitis in HIV-infected patients, whereas dysphagia and odynophagia were independently (*P* < 0.05) associated with candida esophagitis in non-HIV-infected patients. However, heartburn and acid regurgitation were independently (*P* < 0.05) associated with erosive esophagitis in both patient groups. The internal consistency test using Cronbach's α revealed that the 9 symptom scores were reliable in both HIV (α, 0.86) and non-HIV-infected patients (α, 0.85).

This large-scale endoscopy-based study showed that HIV-infected patients have greater GI symptom scores compared with non-HIV-infected patients even after excluding GI-organic diseases. None of the upper GI symptoms predict candida esophagitis in HIV-infected patients, but dysphagia and odynophagia predict candida esophagitis in non-HIV-infected patients. Heartburn and acid regurgitation predict erosive esophagitis in both patient groups.

## INTRODUCTION

Upper gastrointestinal (GI) symptoms are common in both HIV and non-HIV-infected patients.^1–3^ In the current era of highly active antiretroviral therapy (HAART), the incidence of many opportunistic or AIDS-defining diseases has been reduced.^4^ Thus, the characteristics of upper GI disease in HIV-infected patients are likely to be similar to those in the general population.^5^

*Candida* esophagitis (CE) and erosive esophagitis (EE), 2 major types of esophagitis, are seen in both HIV and non-HIV-infected patients.^6,7^ A variety of symptoms including heartburn, acid regurgitation, hunger cramps, nausea, early satiety, belching, dysphagia, and odynophagia have been reported to predict esophagitis.^1,8–11^ However, previous studies were not prospective in design, did not use validated scales, or did not exclude GI-organic diseases despite the presence of typical esophageal symptoms suggestive of these diseases.^1,8–11^ Elucidating disease-specific GI symptoms may allow physicians to avoid delays in diagnosis and prevent poor outcomes or overuse of endoscopy, but it remains unclear which symptoms can predict the 2 types of esophagitis among HIV and non-HIV infected patients.

To address this issue, we evaluated 9 specific upper GI symptoms using a 7-point Likert scale on the day of pre-endoscopy, and diagnosed various upper GI diseases by endoscopy in a large number of HIV and non-HIV-infected patients. The aim was to determine whether upper GI symptoms were different between HIV-infected and non-HIV-infected patients, and to investigate symptoms that are predictive of CE and EE in patients with or without HIV infection.

## METHODS

### Study Design, Setting, and Participants

We conducted a hospital-based, prospective, cross-sectional study at the endoscopy unit of the National Center for Global Health and Medicine (NCGM; Tokyo, Japan) between September 2009 and April 2014. NCGM has 900 beds and is the largest referral center for HIV/AIDS in Japan. Inclusion criteria were as follows: (i) age ≥18 years; (ii) Japanese nationality; (iii) continual or severe upper GI symptoms; (iv) screening for GI cancer. In Japan, where there is a high incidence of gastric cancer, endoscopy is frequently performed for gastric cancer screening. Exclusion criteria were as follows: (i) no informed consent obtained; (ii) unknown medication use; (iii) dependent on activities of daily living (ADL); (iv) inability to understand written documents; (v) use of any antifungal drug within 1 month before endoscopy; and (vi) urgent or early endoscopy for acute GI bleeding.

This study was approved by the ethics committee of the National Center for Global Health and Medicine (No. 1440), and written informed consent was obtained from all patients prior to endoscopy.

### Data Sources and Measurement

A detailed questionnaire was completed at the endoscopy unit on the day of pre-endoscopy.^12,13^ Use of a proton-pump inhibitor (PPI) was defined as intermittent or regular administration within 1 month before the interview. All patients underwent serological testing for HIV before endoscopy. CD4 cell counts in the 1 month before or after endoscopy were obtained from the medical records. Information regarding history of HAART was collected from pre-endoscopy medical records.

Upper GI symptoms were evaluated using the modified GI symptom rating scale (GSRS). The modified GSRS consists of the original GSRS (epigastric pain, heartburn, acid regurgitation, hunger cramps, and nausea) plus early satiety, belching, dysphagia, and odynophagia, and assesses the 9 symptoms using a 7-point Likert scale (1, none at all; 2, minor; 3, mild; 4, moderate; 5, moderately severe; 6, severe; and 7, very severe).^13,14^ The reliability and validity of the GSRS in the assessment of functional GI disease are well documented.^15^

### Diagnosis of Upper GI Disease and Candida Esophagitis

A high-resolution scope (GIF-H260, Olympus Corp., Tokyo, Japan) was used for the diagnosis of upper GI disease. Well-trained staff who were blinded to the questionnaire results performed the endoscopy. When abnormal findings were detected on endoscopy, biopsy, or endoscopic mucosal resection was performed. All removed specimens were evaluated by expert pathologists (>10 years’ experience) for making the final diagnoses of upper GI disease. A diagnosis of CE was made if white esophageal plaques detected on endoscopy could not be washed away ^10^ and pathological assessment with hematoxylin and eosin and periodic acid-Schiff staining or culture for *Candida* species confirmed the clinical findings.^12,13^ The diagnosis of EE was based on the presence of circumferential mucosal breaks in the esophagus.^16^ “Organic GI disease” included CE, erosive esophagitis, ulcer, early cancer, advanced cancer, other malignancy, and post GI resection on endoscopy.

### Statistical Analysis

Baseline characteristics were compared using the Mann–Whitney *U* test or Pearson's chi-square test (Fisher's exact test) for quantitative or qualitative variables, respectively.

To determine whether HIV infection is a risk for upper GI symptoms, associations between HIV infection and upper GI symptom scores were analyzed by univariate and multivariate rank ordered logistic modeling after excluding GI-organic diseases on endoscopy.

To identify predictive symptoms of CE, we adjusted for age, sex, and PPI, which were factors significantly associated with both CE and EE in the univariate analysis and were reported to affect upper GI symptoms.^17^ These associations were evaluated after excluding GI-organic disease on endoscopy. We used univariate and multivariate logistic regression models and estimated the odds ratios (OR) and 95% confidential intervals (CI) of each symptoms. Individuals were classified as having positive upper GI symptoms if they scored ≥2 for each item on the GSRS.^18^

To evaluate the reliability of GSRS, we analyzed internal consistency. Cronbach's α was used for measurement of internal consistency of the 9 GSRS items. α Values were interpreted as follows: ≥0.90, excellent agreement; 0.9 > α ≥ 0.80, good agreement; 0.8 > α ≥ 0.7, acceptable; 0.7 > α ≥ 0.6, questionable; 0.6 > α ≥ 0.5, poor; and α < 0.5, unacceptable. *P* < 0.05 was considered statistically significant. All statistical analysis was performed using Stata version 13 software (StataCorp, College Station, TX).

## RESULTS

### Participants

During the study period, 9337 participants who underwent upper endoscopy were met the inclusion criteria. Of these, 3331 participants were excluded in accordance with the exclusion criteria, the remaining 6011 patients who underwent endoscopy and completed the questionnaire were enrolled in the study.

### Baseline Characteristics of HIV and non-HIV-infected Patients

Differences in baseline characteristics between HIV-infected and non-HIV-infected patients are shown in Table 1. The prevalence of CE and EE was 11.2% (48/430) and 12.1% (52/430) in HIV-infected patients, respectively, whereas it was 2.9% (163/5581) and 10.7% (599/5581) in non-HIV-infected patients, respectively. Endoscopy revealed upper GI-organic diseases in 33.4% (2010/6.011) of patients. No significant difference was observed in endoscopy indication, any organic GI disease, EE, esophageal cancer or malignancy, gastric ulcer, advanced gastric cancer, and duodenal ulcer between groups (Table 1). Factors positively associated with HIV infection were younger age, male sex, CE, esophageal ulcer, and gastric malignancy, whereas the factors negatively associated with HIV infection were PPI use, early esophageal cancer, early gastric cancer, post-gastric resection, and duodenal malignancy (Table 1).

**TABLE 1:**
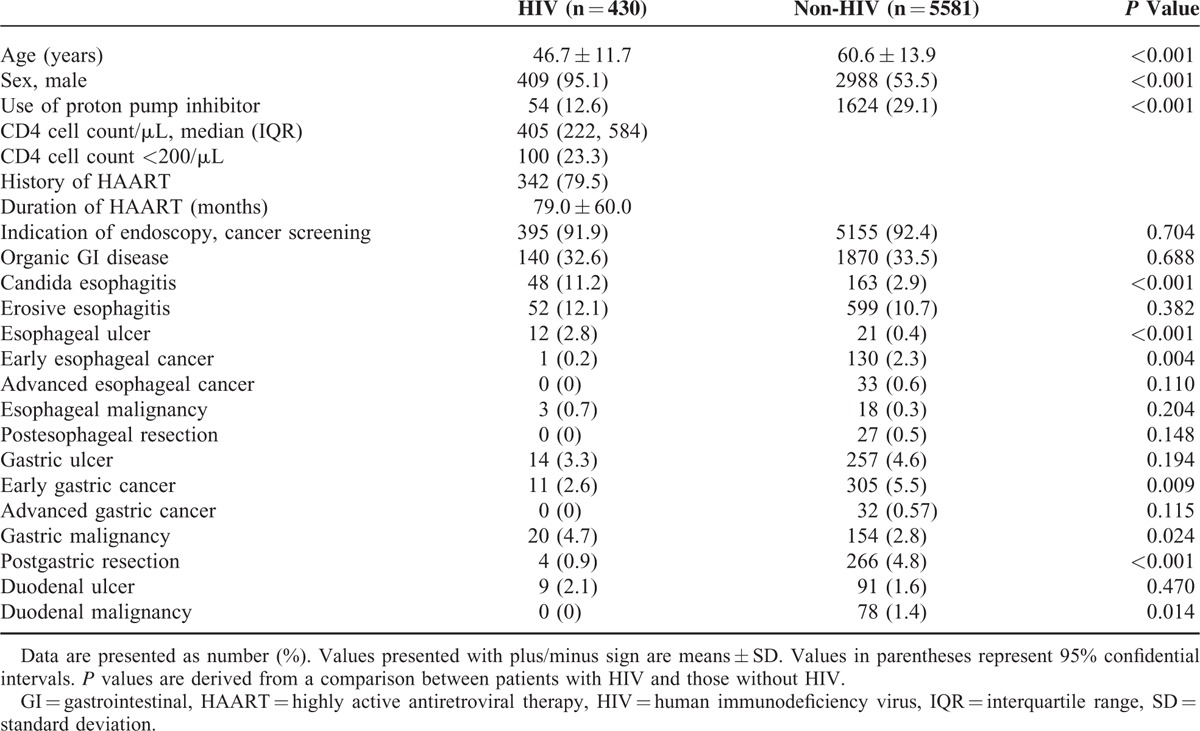
Patient Characteristics in HIV-Infected and Non-HIV-Infected Patients (n = 6011)

### Differences in Upper GI Symptoms Between HIV-Infected and non-HIV-infected Patients

Symptom scores for heartburn, hunger cramps, nausea, early satiety, or odynophagia in HIV-infected patients were significantly higher than those in non-HIV-infected patients after excluding of any GI-organic diseases (Table 2). Multivariate ordered logistic regression analysis adjusted for age, sex, and PPI revealed that the symptom scores for hunger cramps, nausea, early satiety, belching, dysphagia, or odynophagia in HIV-infected patients were significantly higher than those in non-HIV-infected patients (Table 2).

**TABLE 2:**
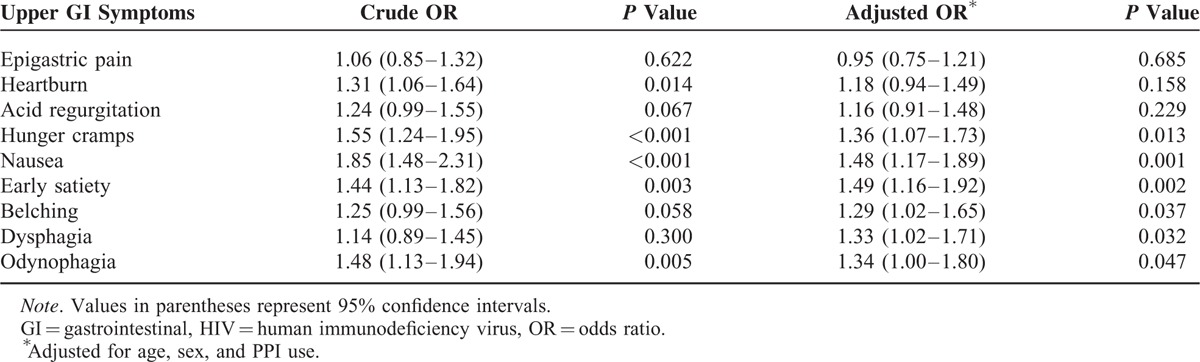
Effect of HIV Infection on the Severity of Upper GI Symptoms After Excluding GI-Organic Diseases (n = 4001)

In HIV-infected patients, we analyzed the effect of low CD4 counts on the severity of upper GI symptoms after excluding of any GI-organic diseases (n = 290). Multivariate ordered logistic regression analysis adjusted for age, sex, and PPI revealed that the symptom scores for early satiety was marginally associated with low CD4 cell count (<200 μL), but other symptoms were not (Table 3).

**TABLE 3:**
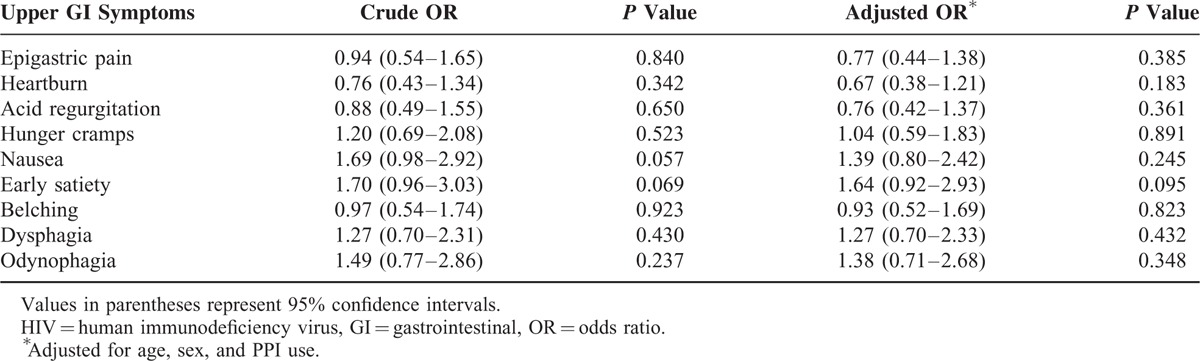
Effect of Low CD4 (<200 /μL) Counts on the Severity of Upper GI Symptoms Among HIV-Infected Patients After Excluding GI-Organic Diseases (n = 290)

### Predictive Symptoms of CE in HIV-infected and non-HIV-infected Patients

There were 211 patients with CE and 3936 patients without any organic GI disease (n = 4147). In multivariate analysis, none of symptoms was positively associated with CE in HIV-infected patients, whereas dysphagia and odynophagia were positively associated with CE in non-HIV-infected patients (Table 4). The other 7 symptoms were not associated with CE (Table 4). Individuals were classified as having positive upper GI symptoms if they scored ≥2 on the modified GSRS. The abovementioned symptoms remained associated with CE in both univariate and multivariate analyses (Supplementary Table 1).

**TABLE 4:**
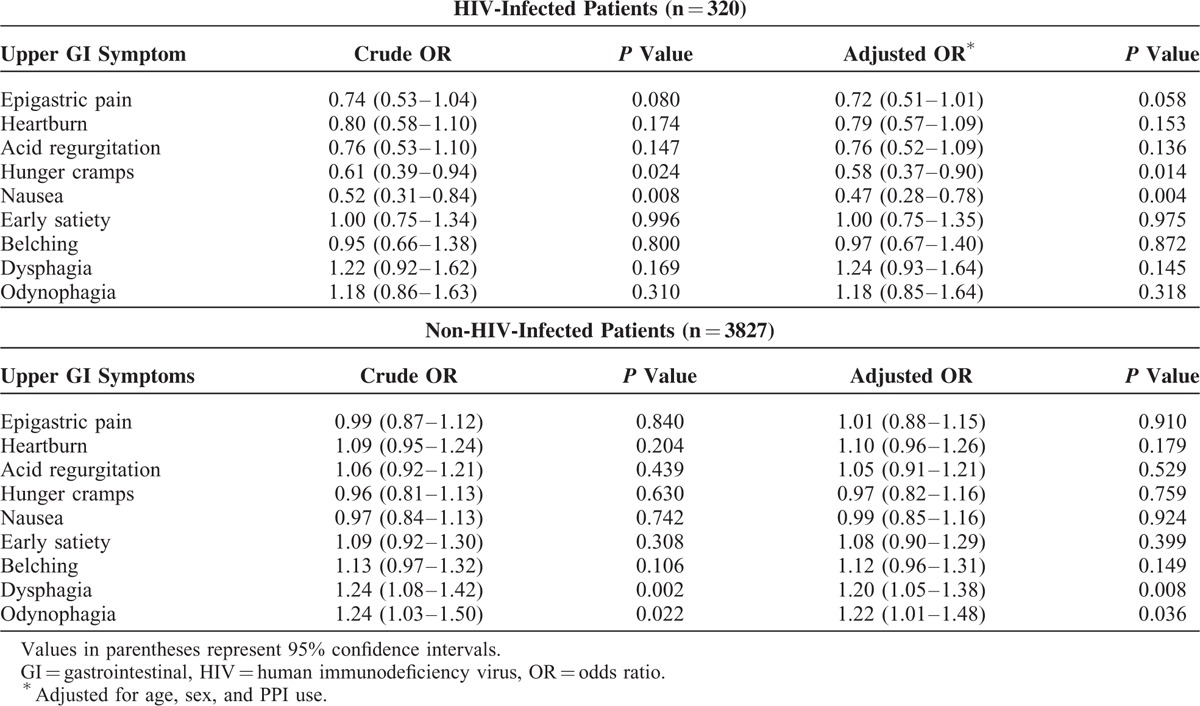
Upper GI Symptom Scores for *Candida* Esophagitis in HIV-Infected and Non-HIV-Infected Patients (n = 4147)

### Predictive Symptoms of Erosive Esophagitis in HIV-infected and non-HIV-infected Patients

There were 651 patients with EE and 3863 patients without any organic GI disease (n = 4514). In multivariate analysis, heartburn and acid regurgitation were positively associated with EE in HIV-infected patients, whereas heartburn, acid regurgitation, nausea, belching, dysphagia, and odynophagia were positively associated with EE in non-HIV-infected patients (Table 5). The other 3 symptoms were not associated with EE (Table 5). Individuals were classified as having positive upper GI symptoms if they scored ≥2 in the modified GSRS. The abovementioned symptoms remained associated with EE in both univariate and multivariate analyses (Supplementary Table 2).

**TABLE 5:**
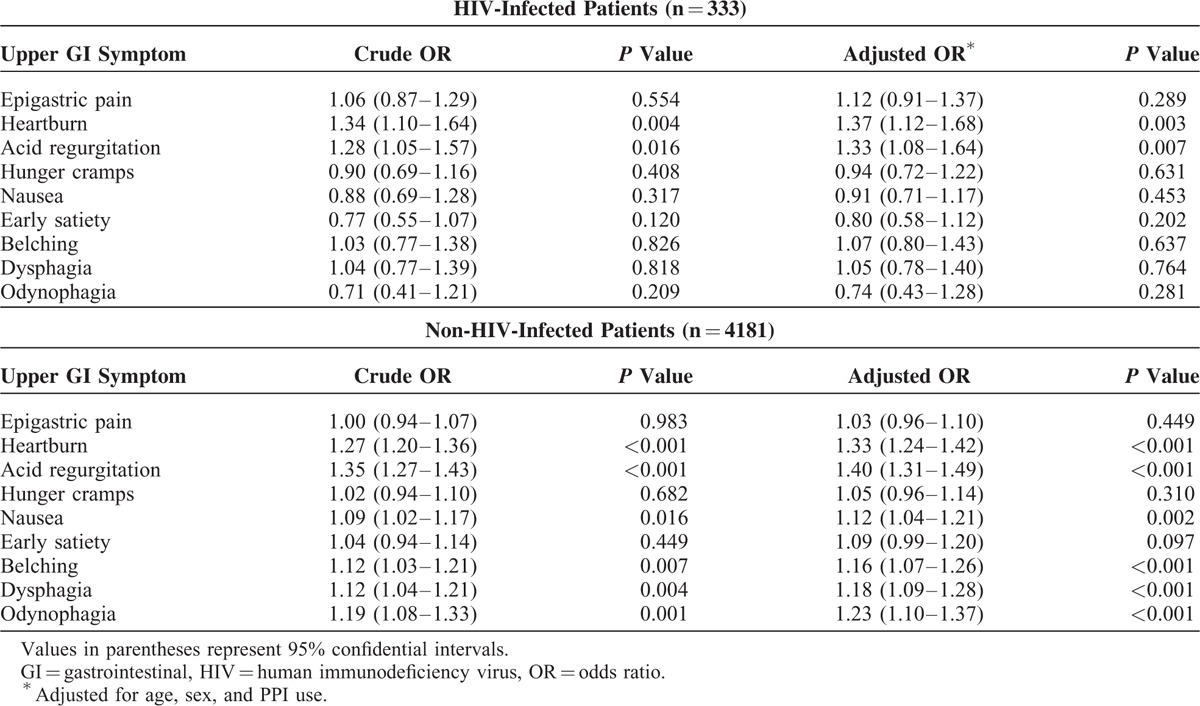
Upper GI Symptom Scores for Erosive Esophagitis in HIV-Infected and Non-HIV-Infected Patients (n = 4514)

### Internal Consistency of Symptom Measurement

The test of internal consistency using Cronbach's α revealed that measurement of GI symptom scores with the 9 items were reliable for both HIV-infected patients (α, 0.86) and non-HIV-infected patients (α, 0.85) after excluding organic disease.

## DISCUSSION

In this study, we investigated 9 upper GI symptoms using a validated 7-point Likert score and we diagnosed CE and EE—2 major esophageal benign diseases^10,19^–using endoscopy. First, multivariate analysis adjusted for age, sex, and PPI revealed that HIV-infected patients had higher upper GI symptom scores than non-HIV-infected patients even after excluding GI-organic diseases. Second, dysphagia and odynophagia were independently associated with CE in non-HIV-infected patients, whereas none of the upper GI symptoms were positively associated with CE in HIV-infected patients. Third, we found that heartburn and acid regurgitation were positively associated with EE in HIV-infected patients, whereas heartburn, acid regurgitation, nausea, belching, dysphagia, and odynophagia were positively associated with EE in non-HIV-infected patients.

The presence of HIV infection increased the severity of hunger cramps, nausea, early satiety, belching, dysphagia, and odynophagia. The reason for this remains unclear, but 1 possible explanation is upper GI dysmotility in HIV-infected patients. Konturek et al ^20^ found that HIV-infected patients with no morphologic changes in the upper GI tract on endoscopy had an abnormal gastric emptying rate, which suggests that upper GI dysmotility causes various upper GI symptoms in the absence of findings on endoscopy in HIV-infected patients. Another possible explanation is medication use. Nausea can occur with use of any protease inhibitor but is more common with zidovudine and didanosine among the nucleoside reverse transcriptase inhibitors.^21^ Indinavir is also associated with esophageal reflux (∼3%).^21^ We did not record the medications of HAART because HIV-infected patients often use multiple medications and frequent changes are made to their medication regimens. In addition, our study showed no significant association between upper GI symptoms and low CD4 cell counts. One possible reason for this is that we used HAART drugs to HIV-infected patients whose CD4 cell count were relatively high according to the guideline.^22^

The information on upper GI symptoms predictive of CE is scarce. Some case series or retrospective studies showed an association between dysphagia or odynophagia and CE in non-HIV-infected patients.^10,23^ Baehr et al^10^ conducted a meta-analysis of 57 reports and found that 63% of CE patients presented with dysphagia or odynophagia and that these were the most frequent symptoms of CE among oral lesions, nausea/vomiting, abdominal pain, weight loss, fever, cough, diarrhea, and rash. Yakoob et al^23^ conducted a retrospective endoscopy-based study and found that prevalence of retrosternal discomfort, dysphagia, and epigastric symptoms was 39.3%, 25.4%, and 35.3% in CE patients, compared with 30.3%, 19.7%, and 50% in non-CE patients, respectively. Although previous studies were small in scale, retrospective, did not use the validated symptom scale to quantitatively assess symptom severity, or excluded organic GI disease, their results are supported by our findings that dysphagia and odynophagia are symptoms predictive of CE in non-HIV-infected patients. The reason why only dysphagia and odynophagia are predictive of CE remains unclear, but a possible explanation is impairment of secondary peristalsis of the esophageal mucosa. Secondary peristalsis is a pressure wave of the esophagus that is triggered by esophageal distention.^24^ Schoeman et al showed that patients with nonobstructive dysphagia commonly had defective secondary peristalsis in response to esophageal distension with boluses of air and water.^25^ In CE patients, *Candida* species cover the esophageal mucosa and cause inflammation of the esophageal mucosa, which may possibly decrease the sensitivity to distension-induced secondary peristalsis and esophageal motility, leading to dysphagia and odynophagia. We found that none of the upper GI symptoms were positively associated with CE in HIV-infected patients. There have been few studies investigating upper GI symptoms and the presence of organic disease on endoscopy in HIV-infected patients; in particular, not many were prospective in design or had used a validated symptom scoring system. Corley et al^1^ investigated the association between 14 upper GI symptoms and the presence of organic disease on endoscopy in HIV-infected patients using a 5-point Likert scale in a questionnaire-based study, and found that none of the symptoms independently predicted upper GI disease, a finding which is supported by our results. Interestingly, they also found that the frequency and severity of upper GI symptoms had negative correlations with the presence of organic diseases. This study and our study suggested that HIV-infected patients have various symptoms without organic diseases. The reason why none of the upper GI symptoms predicted CE in HIV-infected patients remains unknown, but we hypothesize that the nonspecificity of the symptoms may make it difficult to correlate particular symptoms with specific endoscopic abnormalities.

Multivariate analysis showed that heartburn and acid regurgitation can predict EE in both HIV-infected and non-HIV infected patients. Several studies have investigated the association between upper GI symptoms and EE in non-HIV-infected patients.^9,11^ Okamoto et al^9^ found that heartburn (OR, 2.46), odynophagia (OR, 1.36), and acid regurgitation (OR, 1.20) were independently associated with EE by multivariate analysis. Locke et al^11^ found that heartburn frequency, acid regurgitation, and dysphagia were associated with EE. Although these studies investigated only 3 or 4 upper GI symptoms and had not excluded organic GI disease, their findings are in agreement with ours for non-HIV-infected patients. However, symptoms predictive of EE in HIV-infected patients have not been examined, although EE was common in not only non-HIV-infected patients (10.7%) but also HIV-infected patients (12.1%) in our study. In the present era of HAART, the prevalence of opportunistic diseases has decreased while that of non-opportunistic diseases has increased.^4,5^ Moreover, PPI is commonly used in the treatment of EE, which interacts with some medications in HAART.^26^ Therefore, management of EE is becoming more important in HIV-infected patients.

It is controversial whether performing endoscopy for diagnosis is appropriate for patients who have dysphagia, odynophagia, heartburn, or acid regurgitation, which are predictive symptoms of esophagitis. The current guidelines recommend empiric therapy with antifungal drugs or PPI for patients who are initially suspected of having esophagitis.^27,28^ Therefore, we believe that endoscopy is appropriate for patients whose symptoms do not improve after empirical treatment to rule out other etiologies or to investigate the severity of esophagitis.

This study had several strengths. First, this was a large, prospective endoscopy-based study that enabled us to evaluate upper GI symptoms with adjustment for cofounders and exclusion of organic GI disease on endoscopy. Second, we confirmed the internal consistency of 9 GI symptoms in both HIV-infected and non-HIV-infected patients and found that they were reliable items. Third, all patients underwent the HIV serological test before endoscopy. However, this study has limitations. First, we did not assess psychological factors, which are closely associated with functional dyspepsia. Second, this is not a population-based study so selection bias was present. Third, although no significant difference was observed in endoscopy indication or the prevalence of organic GI diseases between HIV-infected and non-HIV-infected patients, the comparison between the small number of patients in the HIV group (n = 430) and the large number of patients in the non-HIV group (n = 5581) may lead to statistical bias.

In conclusion, this large-scale, endoscopy-based, prospective study demonstrated that severity of heartburn, hunger cramps, nausea, early satiety, belching, dysphagia, and odynophagia in HIV-infected patients was significantly greater than those in non-HIV-infected patients. None of the upper GI symptoms predicted CE in HIV-infected patients, whereas dysphagia and odynophagia predicted CE in non-HIV-infected patients. Heartburn and acid regurgitation predicted EE in both HIV-infected and non-HIV infected patients.
